# Public biofoundries as innovation intermediaries: the integration of translation, sustainability, and responsibility

**DOI:** 10.1007/s10961-023-10039-5

**Published:** 2023-11-06

**Authors:** Andrew Watkins, Adam McCarthy, Claire Holland, Philip Shapira

**Affiliations:** 1 Manchester Institute of Innovation Research, Alliance Manchester Business School, The University of Manchester, Manchester, M13 9PL UK; 2 Institute for Manufacturing, University of Cambridge, Cambridge, CB3 0FS UK; 3 School of Public Policy, Georgia Institute of Technology, Atlanta, GA 30332-0345 USA

**Keywords:** Innovation intermediaries, Public biofoundries, Engineering biology, Translation, Sustainability, Responsibility, L30, O32, O32, O33, O38, Q57, R10

## Abstract

The emergence and evolution of engineering biology, and its potential to address multiple global challenges is associated with the rise of biofoundries. These innovation intermediaries are facilities that employ advanced automation and computational analytics to accelerate engineering biology applications. Yet, for biofoundries to fully achieve their promise of generating applications that address grand societal challenges, they need to meet three key challenges: translation of research technology and its commercialization, attention to sustainability, and responsible innovation. Using web content analysis and interviews, this paper explores the functions and capabilities undertaken by existing public biofoundries, the extent to which they address these three challenges, and opportunities and models for enhancement. We also probe the roles undertaken by three other contrasting types of innovation intermediaries to identify practices and opportunities for integration and partnering with public biofoundries. We find that public biofoundries exhibit relatively strong capabilities for research translation, whereas efforts toward sustainability and responsibility are generally less prominent. For biofoundry enhancement, we propose an organisational model based on external partnering where public biofoundries are positioned as intermediaries within regional innovation systems. The framework put forward is reproducible and could be used in other contexts for assessing innovation intermediary organisational functions and capabilities toward meeting societal challenges.

## Introduction

Efforts are increasing to develop bioeconomies where renewable biological resources (including crops and microorganisms) and residual wastes are used to sustainably produce chemicals, consumer and industrial goods, energy, and food (OECD, 2009; NASEM, 2020). A technology that is being positioned to facilitate bioeconomy development is engineering biology, defined here as “the application of rigorous engineering principles to the design of biological systems” (RAE, 2019: 3). Combining synthetic biology, information technology, and automation with an orientation towards applications, engineering biology is positioned as a key driver of new bioeconomies, promising solutions to address climate change, sustainable biofuels, food security, health, and other societal aims (French, 2019; Varjani et al., 2018; McKinsey, 2020).

The growing body of literature that discusses engineering biology’s aims and capabilities indicates that realising engineering biology’s potential, particularly in addressing global societal challenges, hinges on the ability of both the engineering biology community and related public policy to address and meet three interrelated challenges. The first of these is the challenge of *translation* itself in that engineering biology requires a mix of ‘beyond-the-lab’ capabilities for application development, scale-up, and commercialisation (Clarke & Kitney, 2016; Kitney & Freemont, 2012). A second challenge is s*ustainability.* It is often assumed that engineering biology is more sustainable than incumbent applications that use petrochemicals, but this is not necessarily the case when the full range of economic, societal, and environmental aspects are considered (Matthews et al., 2019a; Palmeros Parada et al., 2017). A third challenge is that of *responsibility*. Engineering biology, while promising, also generates societal, ethical, and regulatory concerns that should be anticipated and addressed early in innovation processes (Brian, 2015; Pansera et al., 2020).

The turn towards engineering biology and its translational challenges raises insights and opportunities for designing and adapting innovation intermediaries to aid transitions toward sustainable bioeconomies. We define innovation intermediaries as organisations and initiatives that seek to promote innovation through brokerage, agency, or other transitional interventions without directly benefiting from the eventual product or service (Vidmar, 2020). Such actors foster research translation, technology transfer, commercialisation, and regional development by creating and leveraging knowledge linkages, resource sharing, and agglomeration economies (Howells, 2006; De Silva et al., 2018). Innovation intermediaries are typically publicly sponsored, although they may also have private sector partners (Rossi et al., 2021). Innovation intermediaries in engineering biology include bio-business incubators, bio-scaleup facilities, and participatory bio-science facilities. We suggest they represent a distinct class of intermediaries in that they offer not only ‘dry’ space for computing and office functions but also ‘wet’ lab space for process experimentation, scale-up, and pilot production.

An emerging innovation intermediary in the engineering biology space is the *biofoundry*. Often affiliated with and located in close proximity to universities and related innovation agglomerations, a biofoundry is a highly integrated and automated facility that employs a Design-Build-Test-Learn (DBTL) approach (Holowko et al., 2021), involving high throughput processes (i.e. processes that utilise software, robotics, and liquid handling devices) to rapidly design, build, test, and translate genetically reprogrammed organisms for engineering biology applications, often employing “machine learning and AI approaches for optimisation or potentially for discovering entirely novel design options” (Clarke, 2020: 34). Biofoundry automation of these processes seeks to improve quality, reproducibility and speed (Jessop-Fabre & Sonnenschein, 2019). Biofoundries consist of a mixture of public, private and semi-public organisations, each serving a range of functions in the emerging engineering biology innovation ecosystem (Hillson et al., 2019). Some private engineering biology companies have established biofoundries to increase throughput and process integration primarily for their own R&D and commercialisation objectives (Lesaffre, 2022; Ginkgo Bioworks, 2023). In this study, we focus on non-commercial biofoundries established with public funding which, while varying in terms of proficiencies and focus, typically promise a combination of industrial and broader societal benefits. This includes contributions to regional innovation systems of private and public actors and networks that develop and diffuse knowledge and skills (Asheim et al., 2016) and local and national economic development through their facilitating roles (Farzaneh & Freemont, 2021; GBA, 2023).

Based on their capabilities and funding mandates, these public biofoundries are seemingly well-positioned to address engineering biology’s challenges of translation, sustainability, and responsibility. The public biofoundry community recognises these prospects, but also acknowledges that realising this potential requires a re-thinking of how biofoundries integrate key facility capabilities and how they network and collaborate both with each other and the broader engineering biology community (Hillson et al., 2019). An international association, The Global Biofoundry Alliance (GBA) was founded in 2019. The GBA supports the development and promotion of public biofoundries, and aims to enhance collaboration and communication among biofoundries toward common operational and technological challenges as well as collaboration on grand societal challenges, particularly those related to environmental sustainability and global health (GBA, 2023).

This paper explores the extent to which public biofoundries, individually and collectively, are currently positioned and capable to address the three main translational challenges of engineering biology. We suggest that it is useful and important to investigate biofoundries given their key role in the development of engineering biology, which is a strategic priority technology for many countries (OECD, 2021), and because they represent a new example of efforts to integrate translation, sustainability, and responsibility challenges in research and innovation. We explore three interrelated research questions. First, what are the main intermediary functions and attributes of current public biofoundries? Second, to what extent are public biofoundries currently capable and positioned in terms of addressing the three key challenges of translation, responsibility, and sustainability? Third, how might biofoundries be enhanced, from a functions and capabilities perspective, toward addressing the three main challenges? To answer these questions, we develop a corresponding typological framework and use it to perform a web-based analysis of a set of biofoundries, assessing capabilities, functions, and opportunities for integration. We also apply our framework to examine other innovation intermediaries in the engineering biology space, looking at practices, complementary capabilities, and opportunities for collaboration. In the following sections, we discuss our conceptual approach and how we operationalise it, after which results and findings are presented.

## Conceptual approach

### Innovation intermediaries and their activities

The development of firms and applications in and across new technology fields (such as information technology or biotechnology) is invariably aided by decades of publicly led initiatives aimed at catalysing research and innovation activity. Locations where research and innovation thrive typically host a range of innovation intermediaries – including public research organisations, university technology centres, technology brokers and diffusion agents (Howells, 2006). Early innovation intermediaries included science parks, incubators, and technology transfer and industrial extension offices, while more recent developments involve accelerators, and workspaces to promote software-based start-ups, digital innovation hubs and place-based innovation districts (Link et al., 2015; Shapira & Youtie, 2017; Clayton et al., 2018; Crișan et al., 2021; Rossi et al., 2022).

These varied intermediary types foster innovation through reducing information asymmetries, facilitating interdisciplinary knowledge exchange, promoting university-industry collaboration, providing guidance, resources, and other support services, and undertaking other bridging, catalysing and boundary-spanning activities which accelerate knowledge sharing and commercialisation (Siegel, 2006; Gliedt et al., 2018; Rossi et al., 2022). Innovation intermediaries that are particularly focused on encouraging translation and commercialisation may employ business modelling, spin-out support, and entrepreneurial finance, with some also facilitating pilot production and scale-up. Work by Chesbrough et al. (2006) and others highlight the role of innovation intermediaries in knowledge and technology transfer between partner firms.

From both national and regional perspectives, innovation intermediaries seek to leverage and build innovation capacities in ways that accelerate translational research processes, bringing innovations to market more quickly and avoiding the many pitfalls of the so-called ‘valley of death’ where many innovations languish and ultimately fail (Frank et al., 1996; Beard et al., 2009). As they pursue these objectives, and in designing and delivering an appropriate mix of the intermediary functions discussed above, innovation intermediaries need to address access to resources (including human, financial, and knowledge resources) and respond to the needs and capabilities of firms that are their customers. This can involve overcoming patchiness in absorptive capacities, fostering behavioural and business change as well as technological adoption, and addressing demands and expectations of their stakeholders and sponsors (Cohen & Levinthal, 1990; Russo et al., 2019; De Silva et al., 2022).

### Engineering biology and its core challenges

The promise of engineering biology has been recognised and supported by governments and funding bodies for the past two decades (SBRCG, 2012; EBRC, 2021; Donati et al., 2022; Mao et al., 2021). Current initiatives have emphasised the potential that engineering biology has for ushering in more sustainable societies and lessening the severity and impact of climate change (OECD, 2011; BioMADE, 2021; Horizon Europe, 2022). For example, a 2019 report by the UK’s Royal Academy of Engineering spoke of engineering biology as:

contributing to economic activity and sustainable and resource-efficient solutions to the societal challenges faced in food, chemicals, materials, water, energy, health and environmental protection. Harnessing the capabilities of organisms, processes and mechanisms that exist in nature, and combining this with the incredible advances in areas such as processing power and machine learning presents solutions to problems of all scales – most pertinently in the need for sustainability and reducing emissions (RAE, 2019:3).

However, as with other emerging science and technological fields, the successful economic and business translation of new engineering biology applications to industry is not a foregone conclusion. Engineering biology is a ‘deep tech’ (BCG, 2019), broadly defined by Siegel and Krishnan (2020:8) as a technology that “was impossible yesterday, is barely feasible today, and will quickly become so pervasive and impactful that it is difficult to remember life without.” Furthermore, “deep tech solutions are reimaginations of fundamental capabilities that are faithful to real and significant problems or opportunities, rather than to one discipline.” For engineering biology, as with other deep tech domains, this involves not only radical technological development but also the transformation of existing applications, practices, and business models requiring extensive applied development, substantial capital investment, and a lengthy time to market. Furthermore, engineering biology has the additional uncertainty regarding scale-up in that what works in the lab and at small-scale may prove unstable and unattainable when scaled-up – a necessary final step for commercialisation (BCG, 2019). Relatively few organisations, companies, and regions currently have capacities and resources for engineering biology research translation and commercialisation.

In this way, research translation and commercialisation itself can be viewed as the first key challenge facing the engineering biology community, including related innovation intermediaries (Hillson et al., 2019). Yet, there is more to be addressed. Public policy in support of synthetic and engineering biology has emphasised the long-standing need to carry out research and to develop applications in ways that are responsible both to the environment and society (Serrano, 2007). In a real sense, the promise of environmental sustainability and the need to deliver on this promise in a socially responsible way represent additional challenges for the engineering biology community, beyond technical and translational needs.

This second challenge of environmental sustainability is not unique to engineering biology, with the demand for clean and green technologies receiving growing emphasis in the broad field of technology business incubation (Lamine et al., 2018). However, it is an especially pertinent challenge as engineering biology explicitly seeks to replace many incumbent applications derived from petrochemicals (OECD, 2021). Yet, while environmental sustainability is often assumed in engineering biology, this is not necessarily realised (Matthews et al., 2019b). For example, no biocomposites currently exist that can be considered fully carbon neutral as environmental trade-offs are required for industrial viability (e.g., water and energy requirements, biodegeneration, solvent release, consumables) (Correa et al., 2018). Petrochemical products are already well defined and optimised, from production to delivery, whereas new biobased products are often in the development stage, with immature supply chains (as well as fewer in-built subsidies). Even if these processes are optimised, dependence on imported rather than local feedstocks can negate any competitive environmental benefit that has been conferred using a biobased product over a petrochemical, due to transport emissions.

The third challenge is that of responsible innovation. There are longstanding concerns about how scientific research and technological development can be pursued to mitigate potential societal harms and confer benefits to society (Kranzberg, 1964; Collingridge, 1980). Engineering biology is positioned at the fulcrum of these concerns and debates. With the promise of addressing societal and sustainability goals, there are economic pressures for manufacturing scale-up and market deployment; yet it is a technology that raises substantial societal, environmental, and regulatory challenges (Kemp et al., 2020). Such concerns have been expressed in recent years through the growth of attention to ‘responsible research and innovation’ (RRI). This engages transparent and interactive processes to anticipate and assesses research and innovation’s implications including its ethical, sustainability, and social dimensions (von Schomberg, 2013). An RRI approach addresses this dichotomy by putting forward a framework that emphasises not only anticipation, but also continuous reflection and responsiveness, allowing for the ongoing adjustment of technological development pathways toward societally favourable outcomes. Key to these RRI dimensions is an emphasis on inclusiveness in processes of anticipation and reflection, to engage early (including at design stages) with diverse perspectives and publics (Stilgoe et al., 2013).

The three key challenges that we have described for engineering biology have parallels in other domains. For example, “triple bottom line” concepts that concurrently address economic, environmental, and social performance are long established in discussions of sustainable development (Brundtland, 1987) and business excellence (Elkington, 1998). Similarly, while there are varied frameworks on corporate social responsibility (CSR) and environmental, social and governance (ESG) standards, at their core both concepts embody the notion that corporations and financial institutions in the business world need to be accountable across the full range of their effects, integrating attention to societal and ethical implications, environmental sustainability, and economic impacts into strategies, operations, and the creation of shared value (Latapí Agudelo et al., 2019; Daugaard, 2020. As discussed above, scientific research, innovation, and technology transfer are now increasingly expected to address these three core challenges, as exemplified in the field of engineering biology. Within this growing field, public biofoundries are key intermediaries specifically positioned to span research and innovation in engineering biology. The next section discusses the intermediary functions of public biofoundries and considers how we can understand and operationalise whether these biofoundries are addressing the three key challenges of translation, responsibility, and sustainability.

## Public biofoundries: addressing the three challenges of engineering biology

The concept of the biofoundry, as an automated pilot production and scale-up facility, is relatively recent, with discussion of early examples and projects emerging in the mid-2010s (Eisenstein, 2016). Since then, biofoundries have emerged globally in countries with strong or emerging bioeconomies. While some biofoundries are operated privately, growth is particularly driven by the establishment of public or non-commercial biofoundries as governments promote enhanced access to engineering biology capabilities to spur innovative economic development and address sustainable development goals. In mid-2023, 33 public biofoundries in 12 countries were listed as members of The Global Biofoundries Alliance (GBA, 2023), although the total number of biofoundries worldwide is higher. Many public biofoundries have close affiliations with universities or public research organisations. They provide facilities for researchers to translate their research into commercial applications and for companies to use the biofoundry to develop new applications and products (Holowko et al., 2021).

Public biofoundries add new applied research capabilities into innovation ecosystems, using data analytics, high-throughput analytical equipment, and advanced robotics, aimed at bridging the gap between early stage or basic research and the stages of prototype, scale-up, commercialisation, and full production (the latter typically taking place in separate, dedicated biorefineries and industrial facilities). This requires the biofoundry to perform a second group of functions, to lead in identifying and readying research for translation, i.e. developing a pipeline of potential applications for which the biofoundry’s capabilities are appropriate, and to seek out a range of firms to partner and work with. This contributes to the research pipeline, as well as building up the capabilities of existing and new firms, and either incorporating or linking to pilot and scale-up capabilities (Farzaneh & Freemont, 2021).

Public biofoundries perform typical intermediary functions of research and technology scanning and selection, network building of research communities and industry, and facilitating collaboration and interdisciplinary knowledge exchange. Yet, while biofoundries encompass these intermediary knowledge transfer and networking functions, they are also distinctive in that a substantial part of the innovation process occurs within the biofoundry itself: the biofoundry’s capabilities allow it to carry out actual experiments, prototyping, and in some cases, the scale-up of both potential and actual applications. For both researchers and firms using a biofoundry, the facility acts not as just an intermediary but as a collaborative platform where the developmental pathways of the technology are constructed. This influences not only how the technology will work when it reaches industrial scale but also its sustainability and societal implications, the biofoundry is – and should be – an arena where all three of the key engineering biology challenges are considered. The challenges are discussed in the engineering biology literature and highlighted in policy (for example, OECD, 2011; BioMADE, 2021; Horizon Europe, 2022).

**Fig. 1:**
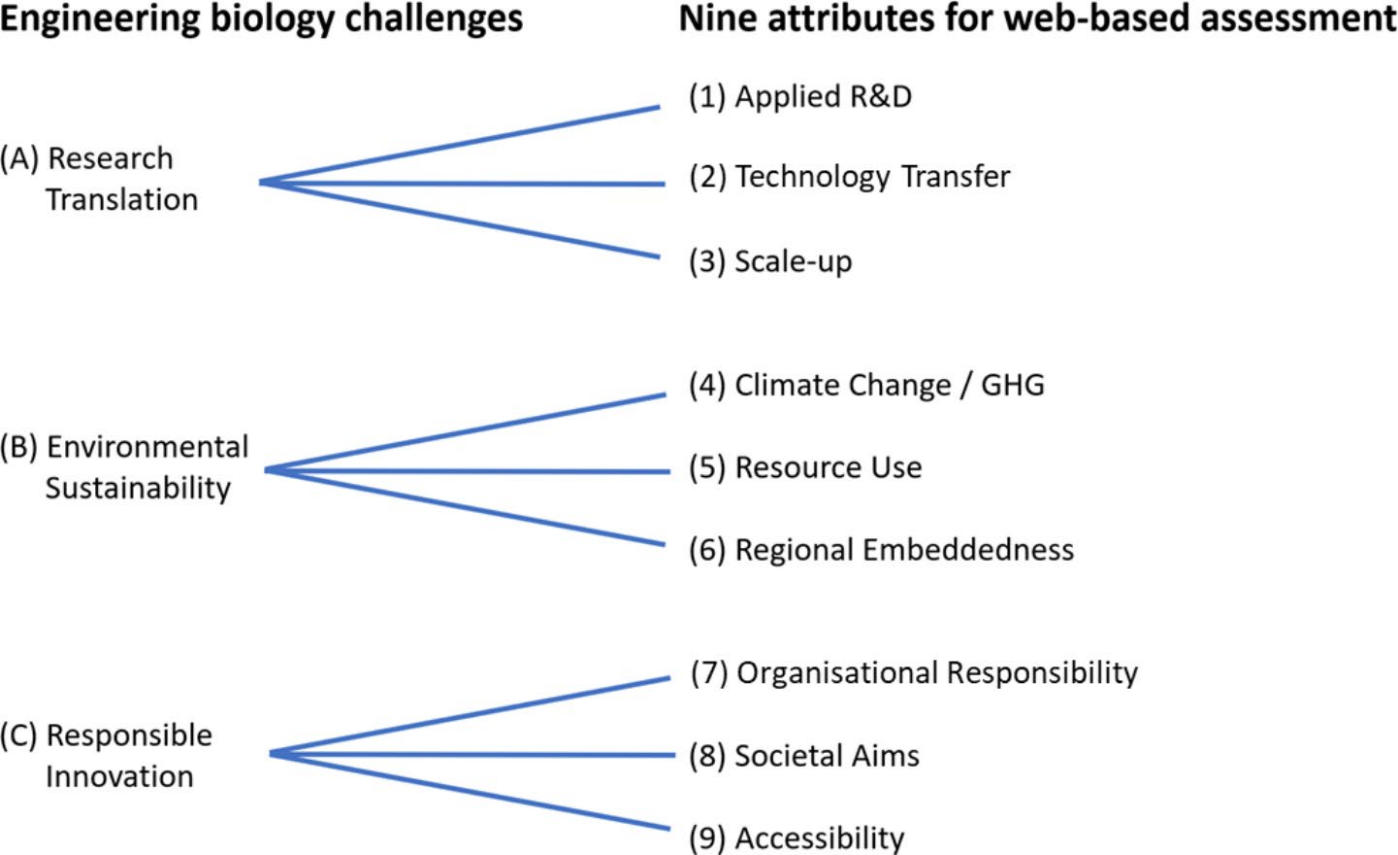
Typology for facility assessment showing the three engineering biology challenges (A-C) and operationalization of assessed attributes (1–9) Source: Authors’ elaboration. The challenges and attributes are discussed in detail in the text

In what follows, we take a closer look at these challenges from the perspective of engineering biology intermediaries. We proceed by operationalising the three challenges, breaking each one down into three further attributes representing indicative functions of the kind that need to be addressed if engineering biology is to perform well against its three key challenges. For each further attribute, we reference engineering biology and biofoundry literature where attention to the attribute is indicated as an essential biofoundry feature. The resulting 3 × 3 framework (leading to 9 factors) is subsequently used for empirical assessment (see also Fig. 1). We acknowledge that it could be possible to highlight additional factors, for example using quantitative measures of economic translation such as added revenues or employment for biofoundry assisted enterprises, or more detailed life-cycle analyses of facilitated biomanufactured products and processes. However, consistent data on such aspects is difficult to obtain for the former and rarely available for the latter. While recognising that there are likely to be limitations, we posit that the nine factors used in our framework provide both breadth and depth to assess performance against the essence of the three challenges across the landscape of biofoundry organisations, are in relevant literature and documentation, and are reasonably assessable in terms of available and accessible sources.

### Operationalising the challenge of research translation

The challenge of research translation for public biofoundries involves several aspects, including identifying promising technologies that not only can be designed, built and tested but also applied for commercial use. This requires that capabilities, partnerships and networks are in place to address intellectual property, licensing, financing, and other business model issues, as well as scale-up competencies to make refinements and to demonstrate that industrial-scale production is feasible. Drawing on this, we suggest that the extent to which biofoundries can successfully address the inherent challenge of research translation will be indicated by capabilities for these three functions:

1. *Applied research.* This refers to the biofoundry’s capabilities to undertake research to apply knowledge for practical use, identify potential applications and actively develop those applications. Performing applied R&D is a fundamental element for biofoundries to serve as intermediaries that bridge upstream fundamental research and downstream industrial production (Hillson et al., 2019; Farzaneh & Freemont, 2021).
2. *Technology transfer.* This refers to the biofoundry’s knowledge exchange, business development and commercialisation capabilities These capabilities may reside in the biofoundry or in an associated tech transfer office or business engagement team. Biofoundry tech transfer functions may also include commercialisation support partnering agreements with companies, facilitating network access to other related actors such as venture capital firms, other financial support sources and market and regulatory expertise, and access to guidance on intellectual property and patenting (NSF, 2023).
3. *Scale-up capabilities.* This refers to whether the biofoundry is established with capabilities that allow for scaling up processes and applications, to significantly increase the batch size of a potential bioengineering product (Holowko et al., 2021). Scale-up capability is critical for commercialisation. Engineering biology applications and products can be challenging to scale up as they will often behave differently when introduced into equipment designed to increase batch size, i.e., what works in the lab does not necessarily work when scaled-up to industrial volumes (Delvigne & Noorman, 2017). Biofoundry scale-up facilities can troubleshoot and optimise these prospective industrial processes.

### Operationalising the challenge of environmental sustainability

Addressing the challenge of environmental sustainability involves explicit attention to the sustainability aspects and limits of bioengineering applications. Varied approaches and tools are available, including simple early sustainability assessments to foster awareness and reflection through to detailed life-cycle assessments along the value chain of feedstock, process, use, and recovery to assess performance and pinpoint opportunities for improvement. Indicative factors include attention to climate change and greenhouse gas aspects and sustainable resource use, especially the use of local feedstocks where available. Since public biofoundries can also be promoted to advance sustainable regional bioeconomies, the level of attention to regional embedding is also important. For a biofoundry, the challenge of environmental sustainability can be operationalised through the following three indicative attributes:

(4) *Climate change/greenhouse gas (GHG) abatement.* This refers to the extent to which a biofoundry is engaged in developing applications that combat climate change by reducing GHG emissions. For example, is the biofoundry advancing biomanufacturing, of improved biofuels or processes that do not involve the use of petrochemicals or is developing engineering biology applications that abate GHG emissions (Carbonell et al., 2020; Thakur & Raghunathan, 2021)?
(5) *Resource use.* Distinct from (4), this category refers to the extent to which a biofoundry is developing applications that not only use less or no petrochemicals, but which use fewer overall resources than incumbent applications and products, particularly in the processes of manufacturing and scale-up. For example, it is possible that some engineering biology processes and applications, while reducing petrochemical use may require significantly more water than incumbent applications (Matthews et al., 2019a).
(6) *Regional embeddedness.* This refers to a facility’s integration into its proximate locality and bioeconomy (Asheim et al., 2016). A regional focus implies a stronger mandate to promote local and regional development and sustainability goals, opportunities for local engagement and input regarding research directions, and linkages with local sustainability perspectives and needs (Isaksen et al., 2022). Regional embedding potentially allows a biofoundry to obtain resources, particularly feedstock from local sources, reducing transport costs and contributing to sustainable regional development (Farzaneh & Freemont, 2021).

### Operationalising the challenge of responsible innovation

For public biofoundries, alongside other innovation intermediaries, the issue regarding responsible innovation is not so much the extent to which a host institution frequently highlights societal missions and responsible innovation (Owen et al., 2020), but rather the extent to which biofoundries tangibly operationalise these values, embed them in their research and innovation processes, and engage with outside stakeholders and publics in their regions and beyond. In this way, responsible innovation can be operationalised through the following indicative attributes:

(7) *Organisational responsibility.* This refers to the extent to which a biofoundry promotes and practices responsible innovation or a similar framework as an organisational mandate (Dixon et al., 2020). For example, does the facility require the organisation and its researchers to follow principles of responsible research and innovation, e.g. anticipation and reflection (Stilgoe et al., 2013) or otherwise indicate that it adheres to ethical research standards (beyond safe laboratory practices)?
(8) *Societal aims.* This refers to whether the biofoundry, through its research, education, and outreach activities, addresses societal needs (Kitney et al., 2019). It is recognised that socially beneficial applications often involve commercial solutions, whereas purely commercial applications can have intended (and unintended) societal benefits. A key marker is whether there is *explicit* attention to societal goals and aims.
(9) *Accessibility.* This refers to a biofoundry’s openness and whether it includes publics and other external stakeholders in research, education, and decision-making activities (Hillson et al., 2019). Does the facility follow inclusive practices and is accessible to a wide range of users, seeking external input from publics and interdisciplinary perspectives?

## Methodology

Our methodological design is consistent with an exploratory study intended to advance concepts, frameworks and early analyses on an emerging topic (Swedberg, 2020), in this case on evolving innovation intermediary roles with a focus on how intermediaries address and integrate responses to key economic, environmental, and societal challenges. For our empirical investigation of the intermediary role performance of public biofoundries, we employed the following multi-step methodological approach.

### Web-based scoping of public biofoundries and sample selection

Biofoundries are not evenly distributed around the world but cluster in a subset of countries that undertake most research in synthetic and engineering biology. These research clusters are primarily in North America (led by the USA), Europe (led by the UK), and East Asia/Oceania (led by China) (Shapira & Kwon, 2018). The available population of public biofoundries reflects this clustering. In early 2021, we reviewed the GBA membership list, which then contained 32 member organisations in four continents (North America, Europe, Asia, and Oceania). We selected 13 biofoundries across nine countries that were publicly funded or accessible to non-commercial users and which presented a range of organisations in terms of capabilities and focus. Of the selected biofoundries, three were in North America with five each from Europe and Asia/Oceania.

For selecting our sample case of biofoundries, purposive expert sampling was used to select case study facilities. This approach uses judgement to identify a non-probability sample to provide information-rich cases that can facilitate exploratory research (Patton, 2002). As a purposive approach, our sample is not designed for empirical generalisation; rather, the aim is to understand the range and diversity of functions operationalised by a set of biofoundries in leading engineering biology research countries.

### Web-based assessment of selected biofoundries

We examined the selected biofoundry study cases by applying our nine-axis typological framework using available online content. We searched facility websites for descriptions of capabilities and activities, including research, education, and outreach, funding, governance, and partners. Analysing web content is now a method frequently used in innovation analysis that has (as with all research methods) strengths and weaknesses (Gök et al., 2015). Features include its unobtrusiveness (useful when information on actions and behaviours is sought) and its openness and accessibility (all study organisations had publicly available websites written in English). At the same time, websites are self-reports and vary in consistency and the number of web pages. Additionally, this method does not reveal sensitive or confidential data, nor does it allow investigation of the financial sustainability of biofoundries.

For each biofoundry, we coded attributes on the nine-axis framework. Two team members independently coded each facility. We acknowledge the potential for bias associated with team member coding and also the potential for differences in interpretation and scoring. In most instances, the assessment scores of both team members were aligned. Where differences arose, the two team members repeated their assessment of that attribute and agreed on a consensus coding score. The following ordinal scale was used: (0) No appearance of the attribute on the website; (1) the attribute appears on the website, e.g. the term ‘societal aims’ appears in the facility overview or list of objectives, but no further description, details, or examples of the attribute are given; (2) the attribute appears alongside text describing some relevant activities; (3) the attribute appears on the website in a formal statement or mandate plus mention of some relevant activities; (4) the attribute appears in a formal statement or mandate and significant number of activities plus relevant projects or applications are evident; and (5) the attribute appears as a formal statement or mandate plus activities plus relevant projects or applications plus evidence of impacts and outcomes. Coding was undertaken in April-July 2021, with analysis (see next section) completed in August-September 2021. The analysis, as shown in Sect. 5, raised questions about the variation in capabilities of the selected biofoundries and how capabilities might be both integrated and enhanced.

### Web-based analysis of other engineering biology innovation intermediaries

We also applied our web-based assessment to selected other types of engineering biology innovation intermediaries, analysing them in the context of the three core challenges. The purpose was not to directly compare such intermediaries to our biofoundry case study sample, but to look for practices within these facilities, exploring complementary capabilities and approaches, and identifying opportunities for partnering. We undertook a scoping process, through literature review and online searching, to identify other intermediaries that would be appropriate for more detailed examination. This process enabled us to identify these three additional categories of intermediaries: (1) *bio scale-up facilities* – intermediaries that undertake scale-up, usually by offering users access to multiple high-volume bioreactors and fermenters. Facilities may offer capabilities for either pilot production (small batch) or commercial scale production (large batch), or both, as well as capabilities for business development (Delvigne & Noorman, 2017); (2) *Bio-business incubators* – facilities that aim to accelerate the growth of engineering biology and other biotech start-up firms, especially through provisioning of resources such as business expertise, physical space, capital, equipment, and networking connections (Grifantini, 2015); and (3) *Participatory Science Facilities (PSFs)* – comprising a diverse range of maker/hacker spaces and community groups in synthetic and engineering biology (also known as DIY-bio groups). They have the common features of physical locations (with laboratory facilities) where people share resources, knowledge, and ideas to engage publics and co-create innovations (Keulartz & van den Belt, 2016). PSFs can also serve roles in incubating new companies.

From this scoping, we purposively selected 15 intermediaries, five each from the categories of business incubators, bio scale-up facilities, and PSFs. For each intermediary, we coded attributes on the nine-axis framework, carrying out the same coding and analysis process as was done with the biofoundry case study sample, but with an emphasis on practices, complementary capabilities, and opportunities for partnering. In this way, the typological framework is not applied for assessment purposes, but for exploring and understanding the functions of these other intermediary types.

### Verification interviews

In the final stage of the study, we carried out interviews with four facilities. This was done not to serve as a separate qualitative study but rather to validate our web-assessment approach, and to also discuss options for capability development, integration, and partnering. Complementing unobtrusive web-based assessments with subsequent case interviews is used in other web-based studies (e.g., Youtie et al., 2021). Our interviews were carried out with managers in one of each organisational type investigated: a biofoundry, a bio-business incubator, a participatory science facility, and a bio scale-up facility. Anonymity in reporting the interviews was promised. Interviews ranged from 30 to 60 min and were conducted online using Zoom between October and December 2021.

## Results

Results are presented in three parts. First, we look at the assessment of the biofoundry facilities. Second, we make comparisons with the other types of innovation intermediaries. Third, we discuss the verification interviews.

### Web-based scores: biofoundries

**Fig. 2:**
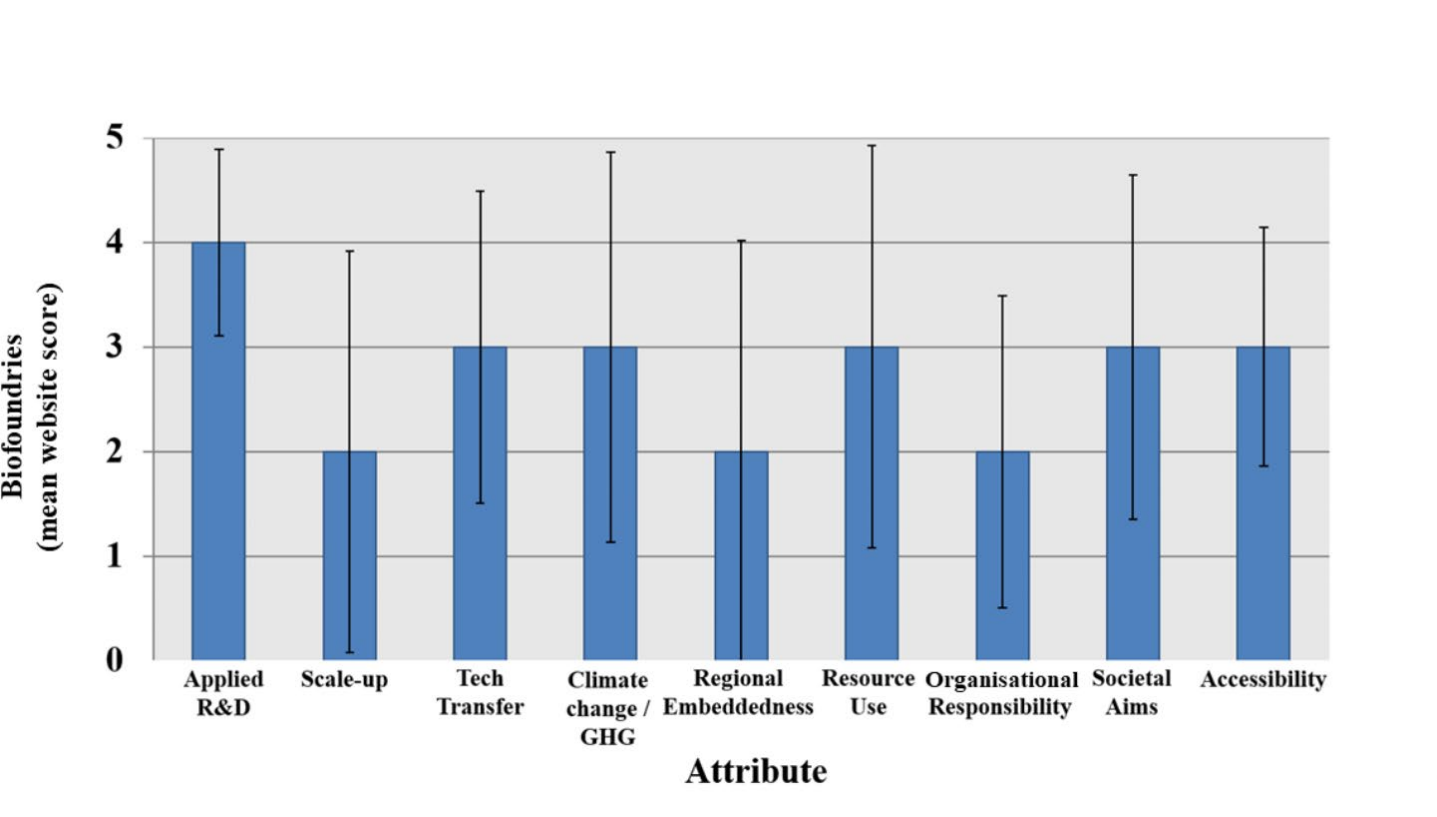
Mean attribute scores (rounded to the nearest integer) for 13 biofoundries. Using a web-based assessment, each facility was scored on the attributes derived from the typological framework, with scores (1–5) determined according to the criteria described in the paper. Error bars indicate standard deviation from the mean. All biofoundries are members of the Global Biofoundries Alliance (GBA).

Biofoundries seek to address the aim ‘to accelerate and enhance both academic and translational research in engineering/synthetic biology’ (Hillson et al., 2019). Yet, our results and analysis showed variations in emphasis (Fig. 2).

For the *challenge of translation*, the case study biofoundries report strengths in both *applied R&D* and *technology transfer*, confirming intermediating roles between basic R&D and translational research. However, an important part of translation is *scale-up*, where, with some exceptions, the biofoundries were weak and did not encapsulate the full process from research to scale-up. One interpretation is that for these facilities, the functions of first selecting and then testing potential applications are relatively strong, but functions that essentially pull such applications toward commercialisation are relatively weak, i.e. despite strong technology transfer programmes, the participation of industry with the aim of commercialisation is lacking; this can have a dampening effect on start-up formation. Another interpretation is that the potential applications selected and tested are not selected for commercial potential and/or are not appropriate in a way that fully utilises the biofoundry’s capabilities. This interpretation aligns with the work of Holowko et al. (2021) and this could be explained by the close connection that most biofoundries have to universities, either being housed within or affiliated with them. Another interpretation is that these biofoundries are translationally limited by their lack of industrial scale-up capabilities, either housed within the facility or realised through connections to external scale-up facilities. Such capabilities, requiring considerable space and infrastructure, may not be compatible with existing facility infrastructure.

In addressing the *challenge of sustainability*, the biofoundries scored relatively well, but with variations by facility and by related subcategories. Average scores for *climate change* and *resource use* were similar. Facilities that focused on one aspect of environmental sustainability tended to also consider the other. Yet, these aspects had high variation, with many facility websites omitting to report on environmental sustainability, whereas for others it was showcased as part of their central mission. Scores for *regional embeddedness* were relatively low. Many biofoundries offer web-based services that can in principle be used from anywhere, which means that their customer or user base may not necessarily be in their immediate vicinity (Hayden, 2014; Jessop-Fabre & Sonnenschein, 2019). On the other hand, most biofoundries are attached to universities, raising expectations of some degree of embeddedness in the local or regional innovation system. Possibly these biofoundries assume that when they are associated with universities, specific mention of regional interactions is not needed.

For the *challenge of responsible innovation*, the average score of 3 for *societal aims* resulted from facility websites either stating their societal and responsible objectives but providing less evidence from actual research examples or impact, or vice versa (i.e., research identified in coding as having potential societal benefit, but little or no stated website discussion). There was a low score, with high variation, for o*rganisational responsibility*, as a few facilities reported strong RRI commitments with evidence of implementation whereas most did not state these aims or mention any actions. Biofoundries reporting attention to RRI tended to be in Europe where this framework has received greater prominence. The middling and low scores in s*ocietal aims* and *organisational responsibility* may reflect the assumption that biobased production inherently delivers more societal benefits than the incumbent modes of production, overlooking the need for coordinated action to achieve such aspirations. (McManus et al., 2015). Facility *accessibility* was an area where most biofoundries garnered mid-range scores. Most facilities primarily support researchers from universities and industry and do not explicitly involve broader publics, although some external educational outreach activities were reported.

**Fig. 3:**
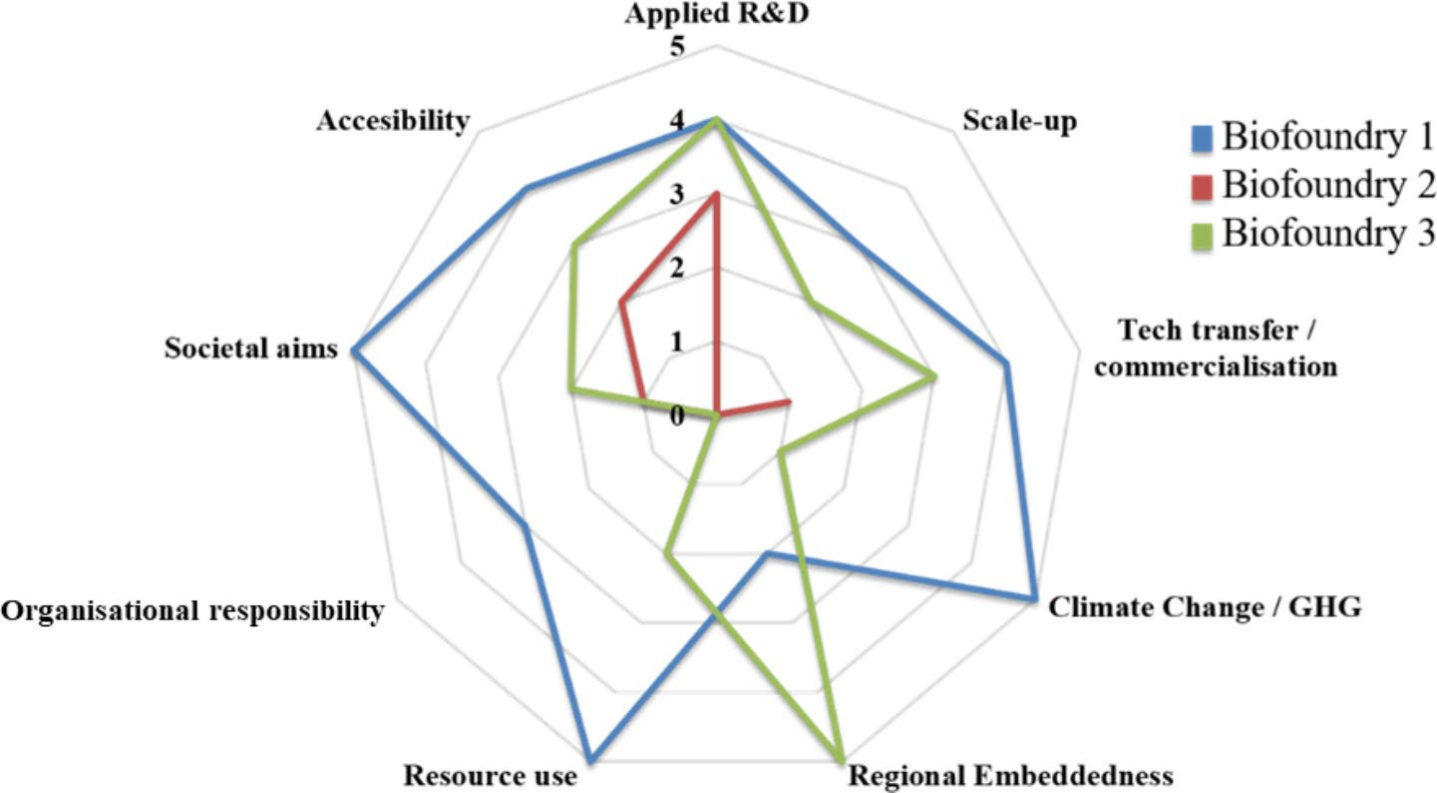
Comparison of typological framework mean scores for three selected biofoundries. Web-based assessment scores (1–5) determined according to the criteria described in the paper. Biofoundry identifiers have been pseudo-anonymised as Biofoundry 1 (Blue), Biofoundry 2 (Red) and Biofoundry 3 (Green)

To highlight variations among biofoundries, we consider scores from three selected GBA members in three different countries. The first of these was chosen due to its well-roundedness, showing strength across most of the attributes addressed. In contrast, the second was chosen due to its relative weakness regarding most attributes addressed. The third was chosen for its strength in terms of regional embeddedness, an attribute assessed as weak for most other biofoundries. We anonymise the results (Fig. 3). Here, Biofoundry 2 shows evidence of applied R&D activities, yet scant mention of activity for other probes, suggesting that the facility identifies with a narrowly targeted mission. This may reflect the implicit assumption that other units of the host university may be performing or are responsible for these other functions. In comparison, other biofoundries present more well-rounded reports. Biofoundry 1 highlights strong applied R&D and technology transfer capabilities. This is complemented by its commitment to societal aims and being relatively open in terms of accessibility. The facility positions itself as a national actor, without substantive mention of regional bioeconomy integration. Again, in contrast to the other two facilities, Biofoundry 3 attained a strong score for regional focus, presenting evidence that its activities were embedded within their regional innovation system. This is reflected in its strong commitment to working with other actors in their science park and local research hospital.

Overall, the typical biofoundry emphasises capabilities in applied research and technology transfer. This is not unexpected given the strong computational and automated capabilities of most biofoundries. However, a particular weakness in scale-up capabilities might be negatively impacting the overall translational effectiveness of most biofoundries. That said, additional translational weaknesses, including those related to scale-up, are challenges in both selecting applications for testing that have high commercial potential, and cementing industrial relations that have strong commitments toward eventual commercialisation. Societal and environmental aims are acknowledged but specific actions are patchier. This could have relevance for, among other things, the selection of potential applications to be tested. On the one hand, a strong commitment to sustainable and responsible applications should drive the selection and subsequent research pipeline for a biofoundry, but on the other hand, commercial potential also needs to be considered. This is not an easy balance to meet. That said, a renewed commitment to regional embeddedness (most biofoundries scoring low in this regard) could provide this balance by connecting sustainable and responsible applications to the resources, capabilities, and commercial needs/potential of a facility’s local/regional bioeconomy – improving a biofoundry’s overall research translation.

Diversity amongst biofoundries is not unexpected in that each operates independently and in its institutional context. Yet, these variations also suggest the nascence of the biofoundry concept, signalling that there are likely opportunities to develop missions to accompany the aims of effective research and translation along with efforts towards societal alignment. As we will see, there are also learning opportunities from the insights gained from other intermediaries. This is especially true for the lowest-scoring categories: scale-up, regional embeddedness, and organisational responsibility.

### Web-based scores: other innovation intermediaries

**Fig. 4:**
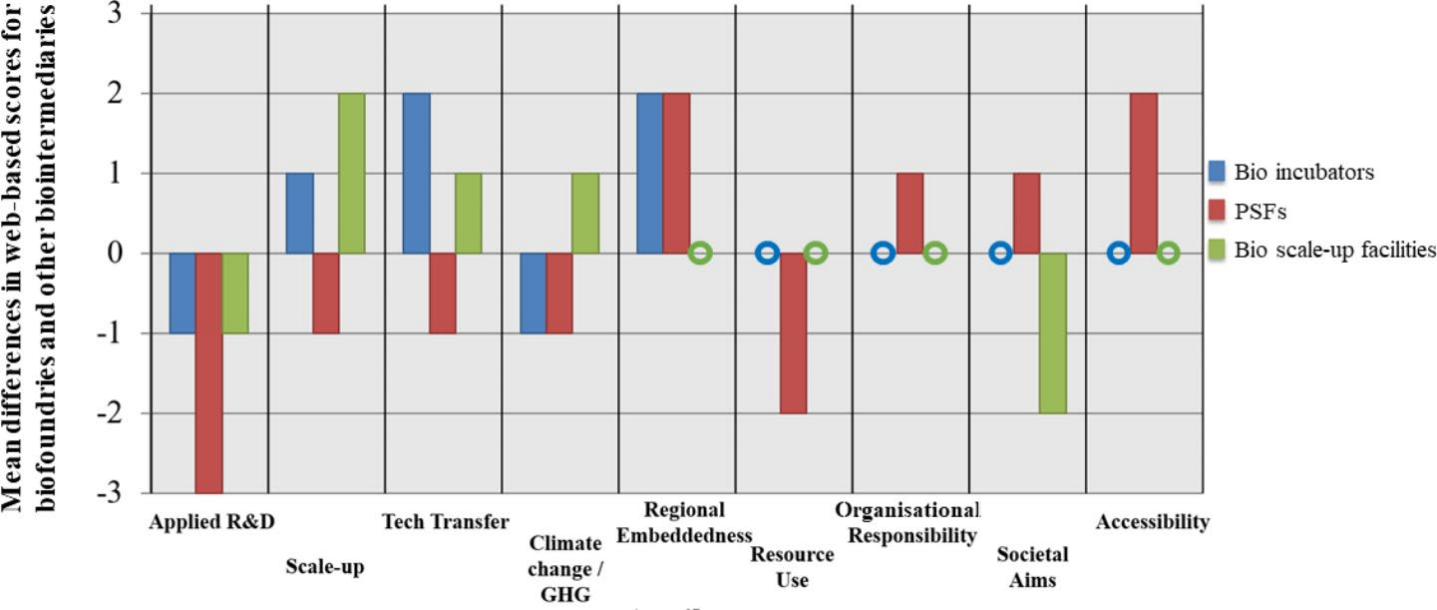
Comparative difference for each attribute between the mean scores for three other intermediary types and the mean biofoundry scores. Differential scores are derived by subtracting the mean biofoundries attribute score from the mean attribute score for three other intermediary types. This was then rounded to the nearest integer. Negative scores indicate that the biofoundries generally outperform the other intermediary type for that attribute. Positive scores indicate that generally the other intermediary type outperforms the biofoundries for that attribute. A zero symbol indicates that biofoundries and the other intermediary type have the same mean scores on that attribute

This section presents results from the other types of innovation intermediaries (bio-scale-up facilities, bio-incubators, and PSFs). We highlight the differences between the average rounded scores for the biofoundries compared with the average rounded scores for the other intermediary types (Fig. 4). The final score represents whether that facility type performed better (positive score) or worse (negative score) than the average biofoundry score for that attribute. Again, it is expected that these intermediaries will perform much differently than the biofoundries: they have different organisational aims and capabilities, but by looking at their various attributes and activities, we look at how they might match up with the capabilities of biofoundries, seeing how they might complement each other. A limitation of our method is that the other studied innovation intermediaries, while engaged in the engineering biology domain, are not necessarily located in the same sub-national region. So, while we can identify variations in activities by intermediary type, we cannot assume or reject an explicit regional division of functions.

Compared with the biofoundries, the bio scale-up facilities and bio-incubators scored higher in technology transfer and scale-up, which is expected as translation is a primary aim of these facilities. Organisational responsibility tended not to score highly for any of the facilities, with an average score of 2 for each facility type except for the PSFs who scored 3. The PSFs also scored relatively highly for accessibility and societal aims. This reflected PSF statements of open accessibility for learning about and doing engineering biology and in decision-making processes via contributing member voting systems and representation on governing boards. PSFs featured work on societally beneficial projects, such as open insulin and creating affordable bioreactors to increase access to engineering biology research (see also Molloy et al., 2018). There were some examples of successful spin-offs from these community spaces, although these were in the minority, potentially because of their limited capabilities in terms of equipment and access to funding. Overall, although PSFs had less translational capabilities compared to the biofoundries, they demonstrated how a facility can be relatively open to public participation while also supporting innovative capabilities (albeit at earlier developmental stages).

Another notable difference is the relatively higher scoring of bio-incubators and PSFs for regional embeddedness. The incubators tended to stress their regional innovation positioning, for example by citing proximate universities and companies or by being part of a science park. There was evidence of purposeful partnerships with regional companies and examples of networks to take advantage of regional knowledge. This was mirrored in the PSFs who stressed their connections to regional innovation systems; they sought to attract interest by offering to share knowledge of experts in local universities or companies. PSFs also highlighted their contribution to the local area. This included providing skills and knowledge for local people, important for emerging bioindustries if they are to positively engage with the region in which they are located.

The overall results for these other innovation intermediaries are not surprising in that they relate to their respective functional and capability niches. These organisational strengths provide opportunities for collaboration with biofoundries, enabling productive partnerships to be formed. Biofoundries could seek to network and collaborate with bio-scale-up facilities to test the scalability of biofoundry-designed applications and have a partner committed to, where applicable, industrial scale-up. Such an arrangement could spur biofoundry efforts towards commercialisation – reinforcing scale-up as a goal – and likely attract more meaningful private investment and industrial involvement. It could also attract more private companies to use the capabilities of the biofoundry. Likewise, partnering with bio-incubator facilities could strengthen a biofoundry’s technology transfer capabilities which may result in improving a biofoundry’s research selection and overall research pipeline.

A further opportunity for functions and capabilities integration comes from a more surprising result of our analysis, mainly the relatively strong regional embeddedness shown by these other innovation intermediaries; they exhibit a strong reliance on networking and collaborating with other actors and publics in their respective local and regional innovation systems (Asheim et al., 2016). Networking and collaboration with these other innovation intermediaries could facilitate a biofoundry’s integration within its own regional innovation system. For example, partnering with spatially proximate bio-incubator and scale-up facilities could not only establish connections with regional SME clusters and/or science parks but also effectively integrate and embed biofoundries within these ecosystems – allowing the biofoundry to reinforce and expand existing innovation networks. Similarly, partnering with, for example, a spatially proximate PSF could enhance a biofoundry’s educational outreach efforts and provides a valuable feedback mechanism for strengthening a facility’s commitment to research and applications that are environmentally sustainable and responsible.

### Interviews and assessment of the web-based approach

The interviews had two primary aims. First, to get feedback from assessed facilities on the accuracy of our web-based approach (discussed below). Second, to explore possibilities for expanding or adapting capabilities identified as currently missing or marginally performed (see Sect. 6).

Overall, interviewees from all four facility types agreed that our assessment of their facilities through their respective websites was generally accurate. For the biofoundry, our assessment of scale-up capabilities was judged as accurate and reflected the challenges faced in using such capabilities toward successful research translation. Responding to a low assessment of regional embeddedness, an interviewee commented that applications appropriate for industrial scale-up were often sent to external scale-up specific facilities (although we were also looking for embeddedness with regional sustainability initiatives). The assessments of commitment to organisational responsibility garnered mixed reactions, with interviewees commenting that we may have underscored facility commitment. However, managers tended to view responsibility from a laboratory perspective (e.g., safe materials handling practices), and not activities that expanded beyond this.

The interviews did highlight some limitations of our approach. For example, an organisation’s website will be customised to its intended audiences, e.g., a biofoundry might highlight its leading-edge engineering biology capabilities to scientists while a bio-incubator will stress its translational and commercial capabilities to potential start-ups. Discussions of societal aims and responsible innovation tended to be limited or absent in both cases. An interviewee commented that a facility’s website may be content constrained if they are part of a larger organisation with multiple programs:

Since we are an entity within a very large public university, we’re only allowed so much flexibility in the design of what we can put on our web pages, because we have to fit within the nomenclature of the university’s web structure. So, I wonder if that could be a limitation of your methodology related to the dimensions. If we were in a standalone entity, where we didn’t have to be part of the university’s website, we would have a very different storytelling mechanism than what you find there.

Within broad feedback that the web-based assessment was mostly accurate, we learned that the assessment approach fared well in assessing facility capabilities. Regarding facility aims, respondents agreed that what we reported concurred with their website statements but had caveats that they pursued some practices (e.g., responsibility) they did not always write about on their websites. That said, the relative effectiveness of our framework for operationalising the three challenges and the feedback from interviewees suggests that the framework itself could be reproduced and applied to assess other biofoundries and a spectrum of innovation intermediaries in the engineering biology space. Furthermore, the framework could also be used to probe such facilities in ways that go beyond what a web-based assessment allows. For example, the framework could be used as a basis for extended semi-structured interviews and/or workshops.

## Discussion: models for capabilities integration

We now turn to our third research question, what enhancements could be made for biofoundries to tackle the three key challenges more effectively? We suggest three exploratory models, informed by our research findings and the literature on innovation intermediaries. Our findings position public biofoundries as performing traditional intermediary functions of network building, the facilitation of collaboration, and the transfer of knowledge between actors, while also generating innovations through in-house research and translational capabilities; these duel intermediary functions, differentiate public biofoundries from most traditional innovation intermediaries, require capabilities grounded in not only particular knowledge and expertise, but capabilities also grounded in specific technology, equipment and related infrastructure. In this way, these models draw on perspectives, identified in the literature, that effective innovation intermediary performance draws on an appropriate mix of internal capabilities and networked embedding into local and regional innovation systems (Howells, 2006; De Silva et al., 2018).

The first model (M1) is a *reconfigured biofoundry* where a more integrated set of capacities are internally developed. In this model, a biofoundry could incorporate an incubator to extend its translational efforts, add early sustainability assessment approaches, seek inputs from publics on research aims and activities, or sponsor a publicly accessible bio-maker/DIY lab. A second model (M2) is a *partnered model* where a biofoundry forms a network with other proximate incubator, scale-up, and PSF organisations, leveraging capabilities for technology transfer, scale-up, and public science, with shared frameworks related to sustainability and responsible innovation. A third model (M3) is a *hybrid model* where a biofoundry combines selected internal capability enhancements with stronger linkages with key partners. For all three models, a key goal is that of integrating various functions and activities into a supportive organisational structure that enables meaningful and concurrent actions to address the three engineering biology challenges.

Undoubtedly, the applicability of these models will depend on conditions that vary by region and country in terms of resources, capabilities, and the portfolios of biofoundries and other organisations already established. M1 (reconfigured) promises an integrated organisational approach and the leveraging of research capabilities, including labs and equipment. However, it requires additional internal resources and a clear strategic objective for integration to address the three key challenges; is likely most appropriate in regions where other intermediaries performing those functions are not apparent. M2 (partnered) builds upon existing regional capabilities and is cost-effective, although additional coordinating resources would be necessary. Its limitations might be the potential disconnection of functions and facilities. It is a model that could be appropriate where a region has multiple, diverse intermediaries, although it would require long-run strategic intent to collaborate among partnered organisations. M3 (hybrid) allows a pragmatic approach that leverages external capabilities where they are available and internal development where they are not. It would require added resources for capability development (although less than for M1) and coordination, as well as intent among collaborating partners. It is perhaps a model that is appropriate for regions with a mixed but not extensive set of existing intermediaries. It is also possible that another engineering biology intermediary within the region is better placed (and resourced) to add capabilities and lead partnership coordination across the three key challenges.

As shown through our web-based assessments, and also mentioned in interviews, most biofoundries do not have industrial scale-up capabilities. This confirms other evidence on the lack of industrial scale-up capability and limited external scale-up options as a major impediment toward meeting engineering biology’s translational challenge (Siegel & Krishnan, 2020; Farzaneh & Freemont, 2021). Further, bringing industrial scale-up capabilities into most existing biofoundries could be difficult in that most biofoundries do not have the physical space, related infrastructure, and expertise needed for such operations. In this sense, scale-up capabilities are qualitatively different from the current capabilities of most biofoundries, i.e., it goes beyond simply expanding a facility’s physical footprint and into the integration of knowledge and systems. In this context, expanding in-house scale-up capabilities (M1) could be prohibitive, at least in the short term. Another option might be to establish new biofoundries with integrated scale-up facilities which develop linkages to other capabilities (M3). Both options would help to research translation and address integration challenges but would require new efforts to secure policy and funding support for facility development.

At the same time, our interviews indicated that a small subset of biofoundries do have industrial scale up capabilities as well as access to other external scale-up specific facilities. However, these facilities are not well promoted within the biofoundry community and, in the instance of scale-up specific facilities, are not always easily accessible. Scale-up specific facilities, and to some extent bio and other business incubators generally provide their facilities and services to users that have an identified commercial pathway and business model. This tends to favour start-ups and SMEs. Research and applications coming out of biofoundries and affiliated universities, while occasionally resulting in start-ups, often lack commercial positioning, pointing to a potential technology transfer gap. In a broader sense, the interviews highlighted the gaps between current engineering biology research, and the capabilities that both biofoundries and scale-up specific facilities hold. An interviewee representing a bio-incubator, commented:

When we have scale-up projects come in, we often do consider whether or not it’d be worth them speaking to a biofoundry to see if it would help them get to where they want to be with their bioprocessing. But we haven’t, as yet identified a project that would be suited to that [appropriate for a biofoundry]. And I don’t know what that says, really about the need for high throughput genome engineering. We see a lot of projects that don’t come from the mass generation of genomes from genome editing. It is possible that the research community, while close, has not gotten there yet.

Enhancing and building partnerships between biofoundries and scale-up facilities, as well as business incubators, as in (M2), could integrate translational research and effective scale-up. Partnering between biofoundries and other organisations to leverage different capabilities does occur, with interviews indicating much of this collaboration occurs within respective regional ecosystems. It is for this reason that (M2) was identified, through the interviews, as the most practical of the three proposed models. As an interviewee representing a PSF commented:

If you’re trying to integrate these things, it’s easier to start having them initially integrated, rather than trying to add them on afterwards. But if you start with an established capability or function, and start asking them to do this other stuff, their core purpose might still be focused on but the other things might take a backseat. So, a new hybrid organization would be perfect, ideal actually, but not in terms of practicality. Because establishing something new always comes with unexpected problems and barriers that you have to jump over. So this integration of capabilities would be best achievable for current biofoundries, perhaps by partnering with other institutes and the strengthening of those partnerships. Currently, we are loosely related to a biofoundry but perhaps strengthening that connection might be the most practical way to achieve this integration and to build mutual capabilities.

M2 is not without challenges, particularly in bringing together multiple organisations with varied objectives. It was noted that greater user and public accessibility would need to address lab safety and containment concerns. Also, organisations such as PSFs operate with a high degree of openness and independence, so partnering with others would need to uphold organisational autonomy. Similarly, partnering needs to be done in a way that organisational attributes and aims are respected. For example, engineering biology applications developed in university-affiliated biofoundries where societal aims and responsible innovation are emphasised should aim to maintain those attributes as the application is picked up by a partnering industrial scale-up facility.

Our study has focused on public biofoundries. However, some implications can be drawn for biofoundries in private companies. There may be opportunities for public biofoundries to form partnerships (M2) with private biofoundries for joint applied R&D, access to specialised scale-up capabilities, training, or collaborative support for spin-out enterprises. There are also broader insights from our study of public biofoundries. Private biofoundries, embedded in commercial enterprises, may not have public mandates for addressing societal challenges, responsibility, sustainability, or civic accountability. Yet, they may well be operating as part of engineering biology companies that have expressed corporate intent to foster sustainability and responsible innovation in addition to, or as part of, their commercial objectives. Moreover, these broader goals may be embodied in support that these companies have received from private or public funders. Business success, especially in the emerging field of engineering biology, requires simultaneous attention to societal and environmental aspects as well as commercial market opportunities. Private biofoundries, as they reflect on how they are incorporating attention to these aspects, will likely find the framework and findings of this study to be useful. They may also consider which of the three pathways (in-house, partnered, or hybrid) may be most appropriate in building up needed capabilities.

## Conclusions

The growth of engineering biology and its potential to address multiple global challenges, has been accompanied by the rise of biofoundries. These facilities face challenges of translating research into applications that are marketable, environmentally sustainable, and socially responsible. We explored how selected current biofoundries and other innovation intermediaries address these challenges and how activities might be enhanced and integrated going forward.

In addition to examining biofoundry development in emerging engineering biology, our contribution is based on the investigation of new types of intermediary organisations that can integrate technical and innovation capabilities in the context of societal and sustainability challenges. We position the public biofoundry as a distinct type of innovation intermediary that performs traditional intermediary functions (such as applied R&D and lab to industry translation) but which is also challenged to address environmental sustainability and responsible innovation concerns. Much of the extant literature on innovation intermediaries focuses on their lab-to-industry translation and regional innovation roles (Holland et al., 2024). We acknowledge the criticality of these objectives. We also emphasise the importance today for innovation intermediaries to integrate translation with responsibility and sustainability and to foster regional embeddedness that encompasses economic, societal and environmental aspects. We deploy a reproducible typology and methodological approach that can be used to assess intermediary capabilities toward addressing the three challenges. While applied to biofoundries in engineering biology, this typology could be expanded and refined for other sectors and facility types, particularly as an exploratory research tool that can inform subsequent, more in-depth, organisational research and analysis.

We found that the studied public biofoundries perform well in applied R&D and technology transfer, but translation can be stymied by a lack of substantive scale-up capabilities. Biofoundries scored in the mid-range for addressing the challenge of environmental sustainability. Existing biofoundries performed less strongly when it came to addressing the challenge of responsible innovation, scoring in the mid-range for societal aims and accessibility but lower for organisational responsibility. The other intermediaries varied in their performance when matched with biofoundries. Bio-incubators and scale-up facilities performed well for translational research, based on their commercialisation and scale-up capabilities, whereas PSFs were particularly strong in terms of user accessibility. In contrast to the assessed biofoundries, all other intermediary types reported greater regional embeddedness.

Interviews with four organisations, one for each type of facility assessed, confirmed the accuracy of our assessments, although with some caveats. From our web-based findings and the interviews, we put forward three models that, variously, converge the strong applied research capabilities of existing public biofoundries with the accessibility, local engagement and regional leveraging exemplified by PSFs, the technology transfer capabilities of bio-incubators, and the industrial scale-up capabilities of bio-scale-up facilities. Establishing fully comprehensive biofoundries that hold and integrate a wider range of functions, including scale-up capabilities, is costly although there may be ways to better use existing facilities. A pragmatic way forward could be for existing public biofoundries to collaborate closely and purposely with bio-incubators, bio scale-up facilities, and PSFs, especially where regional partnerships are possible.

From a broader perspective, while confirming the importance of intermediaries in innovation, we suggest that innovation intermediaries can be further supported to go beyond the conventional technology transfer missions. In order to do this, innovation intermediaries need to find ways to incorporate attention to sustainability and responsible innovation in research and innovation, including when enabling scale-up. We surface insights from engineering biology and the performance of intermediaries of varied types in addressing challenges of translation, sustainability, and responsibility. Further work along these lines, using web content mining and other methods, would aid understanding of innovation intermediary roles in other emerging technology domains and how these roles might be integrated and enhanced.

## Data Availability

Publicly available web content was used in the study, with the listing of case study organizations and the assessment scores (anonymized) that underpin the findings of this study available in the article and appendix.

## Appendix

**Table A1:** List of 28 case study organisations

Type	Organisation	Location	Country
B	Advanced Biofoundry Shenzhen, Shenzhen Institutes of Advanced Technology (SIAT)	Chinese Academy of Sciences, Shenzen	China
B	Agile BioFoundry	US Department of Energy - National Laboratories (multiple sites)	US
B	Australian Foundry for Advanced Biomanufacturing	University of Queensland, Brisbane	Australia
B	CompuGene	Technical University of Darmstadt	Germany
B	Concordia Genome Foundry	Concordia University, Montreal, Quebec	Canada
B	Earlham Biofoundry	The Earlham Institute, Norwich	UK
B	Edinburgh Genome Foundry	University of Edinburgh	UK
B	Illinois Biological Foundry for Advanced Biomanufacturing (iBioFAB)	University of Illinois at Urbana-Champaign	US
B	London Biofoundry, SynbiCITE	Imperial College London	UK
B	Singapore BioFoundry, Synthetic Biology for Clinical and Technological Innovation (SynCTI)	National University of Singapore	Singapore
B	SKy Biofoundry	Sungkyunkwan University, Seoul	South Korea
B	Tianjin BioFoundry, Tianjin Institute of Industrial Biotechnology	Chinese Academy of Sciences, Tianjin	China
B	VTT (cell factory and biochemicals)	VTT Technical Research Centre of Finland	Finland
I	Bio-Riidl	Mumbai	India
I	MBC BioLabs	San Francisco, CA & San Carlos, CA	US
I	Petri	Boston, MA	US
I	Planet B.io	Biotech Campus Delft	Netherlands
I	Sid Martin Biotech	University of Florida, Gainsville, FL	US
P	Biomaker	Cambridge	UK
P	BOSLab	Cambridge, MA	US
P	Counter Culture Labs	Oakland, CA	US
P	London Biohackspace	London Hackspace	UK
P	Sound BioLab	Seattle, WA	US
S	Bio Base Europe Pilot Plant (BBEPP)	Gent	Belgium
S	Bioeconomy Institute (MSUBI)	Michigan State University, Holland & Lansing, MI	US
S	BioMADE (Bioindustrial Manufacturing Innovation Institute)	University of Minnesota, St. Paul, MN; member facilities in multiple US states	US
S	Industrial Biotechnology Innovation Centre (IBioIC)	Glasgow	UK
S	MicroBioRefinery (MBR)	University of Liverpool	UK

Key: B = biofoundry; I = bio incubator; S = bio scale-up facility; P = participatory science facility or community bio initiative

## Appendix 2. Scoring of web-based assessments of case study organisations (anonymised)

**Table:**

Type	Research translation			Environmental sustainability			Responsible innovation		
	RD	TT	SU	CC	RU	RE	OR	SA	A
B	2	4	4	2	2	0	2	3	4
B	3	1	0	0	0	0	0	1	2
B	4	4	0	0	0	0	2	0	5
B	4	3	2	1	2	5	0	2	3
B	4	3	0	3	4	5	2	2	0
B	4	4	3	5	5	2	3	5	4
B	4	2	0	5	5	5	3	5	3
B	5	0	2	0	0	0	2	5	3
B	5	5	0	2	3	2	1	4	3
B	5	5	4	3	4	3	4	3	3
B	5	4	5	4	4	3	3	4	3
B	5	3	3	4	5	0	0	5	3
B	5	5	5	5	5	0	5	5	2
I	0	5	3	1	2	4	3	2	3
I	2	5	3	2	3	2	2	4	5
I	4	5	4	2	2	4	2	3	2
I	4	5	4	4	4	5	3	5	2
I	4	5	4	1	2	3	0	2	2
P	1	0	0	2	3	5	5	4	5
P	1	3	1	0	0	2	2	4	5
P	4	5	1	0	0	4	2	3	5
P	1	2	2	3	3	3	2	5	5
P	1	2	2	1	1	4	2	5	5
S	2	5	5	2	2	4	0	2	2
S	3	5	4	2	2	4	2	2	3
S	2	5	5	4	4	0	1	2	2
S	3	5	5	4	4	0	5	3	4
S	4	3	3	4	4	3	1	0	2

Note. Organisational type code: B = biofoundry; I = bio incubator; S = bio scale-up facility; P = participatory science facility or community bio initiative. Attributes for web-based assessment: RD = applied R&D; TT = technology transfer; SU = scale-up; CC = climate change/GHG; RU = resource use; RE = regional embeddedness; OR = organisational responsibility; SA = societal aims; A = accessibility. See text for explanation of method and assessment scoring. Organisations are anonymised and listed in random order (different from Appendix Table 1)

## References

[CR1] Asheim, B. T., Grillitsch, M., & Trippl, M. (2016). Regional innovation systems: past – present – future. Chapter 2, 45–62. In: Shearmu, R., Carrincazeaux, C., & Doloreux, D. (eds.), Handbook on the Geographies of Innovation, Chap. 2, 45–62, Cheltenham, UK: Edward Elgar Publishing.

[CR2] BCG (2019). The Dawn of the Deep Tech Ecosystem. Boston Consulting Group. https://media-publications.bcg.com/BCG-The-Dawn-of-the-Deep-Tech-Ecosystem-Mar-2019.pdf

[CR3] Beard, T. R., Ford, G. S., Koutsky, T. M., & Spiwak, L. J. (2009). A Valley of Death in the innovation sequence: An economic investigation. *Research Evaluation*, *18*, 343–356. 10.3152/095820209X481057

[CR4] BioMADE (2021). 4s – social dimensions, BioMADE. Available at: https://www.biomade.org/social-dimensions (Accessed: 20 May 2023).

[CR5] Brian, J. D. (2015). Special perspectives section: Responsible research and innovation for synthetic biology. *Journal of Responsible Innovation*, *2*(1), 78–80. 10.1080/23299460.2014.1001971

[CR6] Brundtland, G. (1987). *Report of the World Commission on Environment and Development: Our common future. United Nations*. Oxford University Press.

[CR7] Carbonell, P., Le Feuvre, R., Takano, E., & Scrutton, N. S. (2020). In silico design and automated learning to boost next-generation smart biomanufacturing. *Synthetic Biology*, *5*(1), ysaa020. 33344778 10.1093/synbio/ysaa020 PMC7737007

[CR8] Chesbrough, H., Vanhaverbeke, W., & West, J. (Eds.). (2006). *Open Innovation: Researching a new paradigm*. Oxford University Press.

[CR9] Clarke, L. (2020). Synthetic biology, engineering biology, market expectation. *Engineering Biology*, *4*(3), 33–36. 36968158 10.1049/enb.2020.0021 PMC9996698

[CR10] Clarke, L. J., & Kitney, R. I. (2016). Synthetic biology in the UK - an outline of plans and progress. *Synthetic and Systems Biotechnology*, *1*(4), 243–257. 29062950 10.1016/j.synbio.2016.09.003 PMC5625736

[CR11] Clayton, P., Feldman, M., & Lowe, N. (2018). Behind the scenes: Intermediary organizations that facilitate science commercialization through entrepreneurship. *Academy of Management Perspectives*, *32*, 104–124. 10.5465/amp.2016.0133

[CR12] Cohen, W. M., & Levinthal, D. A. (1990). Absorptive capacity: A new perspective on learning and innovation. *Administrative Science Quarterly*, *35*, 128–152. 10.2307/2393553

[CR13] Collingridge, D. (1980). *The Social Control of Technology*. Open University Press.

[CR14] Correa, J., Montalvo Navarrete, J., & Hidalgo-Salazar, M. (2018). Carbon footprint considerations for biocomposite materials for sustainable products: A review. *Journal of Cleaner Production*, *208*, 785–794. 10.1016/j.jclepro.2018.10.099

[CR15] Crișan, E. L., Salanță, I. I., Beleiu, I. N., Bordean, O. N., & Bunduchi, R. (2021). A systematic literature review on accelerators. *Journal of Technology Transfer*, *46*, 62–89. 10.1007/s10961-019-09754-9

[CR16] Daugaard, D. (2020). Emerging new themes in environmental, social and governance investing: A systematic literature review. *Accounting & Finance*, *60*, 1501–1530. 10.1111/acfi.12479

[CR19] Delvigne, F., & Noorman, H. (2017). Scale-up/scale-down of microbial bioprocesses: A modern light on an old issue. *Microbial Biotechnology*, *10*, 685–687. 28556613 10.1111/1751-7915.12732 PMC5481528

[CR18] De Silva, M., Howells, J., Khan, Z., & Meyer, M. (2022). Innovation ambidexterity and public innovation intermediaries: The mediating role of capabilities. *Journal of Business Research*, *149*, 14–29. 10.1016/j.jbusres.2022.05.013

[CR17] De Silva, M., Howells, J., & Meyer, M. (2018). Innovation intermediaries and collaboration: Knowledge–based practices and internal value creation. *Research Policy*, *47*, 70–87. 10.1016/j.respol.2017.09.011

[CR20] Dixon, T. A., Curach, N. C., & Pretorius, I. S. (2020). Bio-informational futures. The convergence of artificial intelligence and synthetic biology. *EMBO Reports*, *21*, e50036. 32043291 10.15252/embr.202050036 PMC7054666

[CR21] Donati, S., et al. (2022). Synthetic biology in Europe: Current community landscape and future perspectives. *Biotechnology Notes*, *3*, 54–61. 10.1016/j.biotno.2022.07.003 PMC11446344 39416454

[CR22] EBRC. (2021). *Engineering Biology & materials Science: A Research Roadmap for Interdisciplinary Innovation*. Engineering Biology Research Consortium. https://roadmap.ebrc.org/.

[CR23] Eisenstein, M. (2016). Living factories of the future. *Nature*, *531*, 401–403. 26983542 10.1038/531401a

[CR24] Elkington, J. (1998). Accounting for the triple bottom line. *Measuring Business Excellence*, *2*(3), 18–22. 10.1108/eb025539

[CR25] Farzaneh, T., & Freemont, P. S. (2021). Biofoundries are a nucleating hub for industrial translation. *Synthetic Biology*, *6*, 1. 10.1093/synbio/ysab013 PMC8546609 34712838

[CR26] Frank, C., Sink, C., Mynatt, L., Rogers, R., & Rappazzo, A. (1996). Surviving the valley of death: A comparative analysis. *Journal of Technology Transfer*, *21*, 61–69. 10.1007/BF02220308

[CR27] French, K. E. (2019). Harnessing synthetic biology for sustainable development. *Nature Sustainability*, *2*(4), 250–252. 10.1038/s41893-019-0270-x

[CR28] GBA (2023). Global Biofoundries Alliance. https://biofoundries.org/ [Accessed 16 June 2023].

[CR29] Ginkgo Bioworks (2023). Our foundry brings economies of scale to cell programming. Ginkgo Bioworks Holdings Inc.: Form 10-K 2022, 14–18. 13 March. https://www.sec.gov/ix?doc=/Archives/edgar/data/1830214/000095017023007461/dna-20221231.htm

[CR31] Gök, A., Waterworth, A., & Shapira, P. (2015). Use of web mining in studying innovation. *Scientometrics*, *102*, 653–671. 26696691 10.1007/s11192-014-1434-0 PMC4677352

[CR30] Gliedt, T., Hoicka, C. E., & Jackson, N. (2018). Innovation intermediaries accelerating environmental sustainability transitions. *Journal of Cleaner Production*, *174*, 1247–1261. 10.1016/j.jclepro.2017.11.054

[CR32] Grifantini, K. (2015). Incubating innovation: A standard model for nurturing new businesses, the incubator gains prominence in the world of biotech. *IEEE Pulse*, *6*, 27–31. 10.1109/MPUL.2015.2476542 26583888

[CR33] Hayden, E. C. (2014). The automated lab. *Nature News*, *516*, 131. 10.1038/516131a 25471888

[CR34] Hillson, N., Caddick, M., Cai, Y., Carrasco, J. A., Chang, M. W., Curach, N. C., & Freemont, P. S. (2019). Building a global alliance of biofoundries. *Nature Communications*, *10*, 1–4. 10.1038/s41467-019-10079-2 PMC6506534 31068573

[CR35] Holland, C., McCarthy, A., Ferri, P., & Shapira, P. (2024). Innovation intermediaries at the convergence of digital technologies, sustainability, and governance: A case study of AI-enabled engineering biology. *Technovation*, *129*, 102875. 10.1016/j.technovation.2023.102875

[CR36] Holowko, M. B., Frow, E. K., Reid, J. C., Rourke, M., & Vickers, C. E. (2021). Building a biofoundry. *Synthetic Biology*, *6*(1), ysaa026. 33817343 10.1093/synbio/ysaa026 PMC7998708

[CR37] Horizon Europe (2022). Industrial biotechnology approaches for improved sustainability and output of industrial bio-based processes, Funding & tenders. Available at: https://ec.europa.eu/info/funding-tenders/opportunities/portal/screen/opportunities/topic-details/horizon-cl6-2023-zeropollution-01-5 (Accessed: 20 May 2023).

[CR38] Howells, J. (2006). Intermediation and the role of intermediaries in innovation. *Research Policy*, *35*, 715–728. 10.1016/j.respol.2006.03.005

[CR39] Isaksen, A., Trippl, M., & Mayer, H. (2022). Regional innovation systems in an era of grand societal challenges: Reorientation versus transformation. *European Planning Studies*, *30*(11), 2125–2138. 10.1080/09654313.2022.2084226

[CR40] Jessop-Fabre, M. M., & Sonnenschein, N. (2019). Improving reproducibility in synthetic biology. *Frontiers in Bioengineering and Biotechnology*, *7*, 18. 30805337 10.3389/fbioe.2019.00018 PMC6378554

[CR41] Kemp, L., Adam, L., Boehm, C. R., Breitling, R., Casagrande, R., Dando, M., Djikeng, A., Evans, N. G., Hammond, R., Hills, K., Holt, L. A., Kuiken, T., Markotić, A., Millett, P., Napier, J. A., Nelson, C., ÓhÉigeartaigh, S. S., Osbourn, A., Palmer, M., Patron, N. J., Perello, E., Piyawattanametha, W., Restrepo-Schild, V., Rios-Rojas, C., Rhodes, C., Roessing, A., Scott, D., Shapira, P., Smith, S. C., Sundaram, R. D., Takano, L. S., Uttmark, E., Wintle, G., B., Zahra, N.B., & Sutherland (2020). W.J. Bioengineering horizon scan 2020. Elife, 29: e54489. 10.7554/eLife.54489 PMC7259952 32479263

[CR42] Keulartz, J., & van den Belt, H. (2016). *DIY-Bio - economic, epistemological and ethical implications and ambivalences* (12 vol., p. 7). Life Sciences, Society and Policy. 10.1186/s40504-016-0039-1 PMC4884673 27237829

[CR44] Kitney, R., Adeogun, M., Fujishima, Y., Goñi-Moreno, A., Johnson, R., Maxon, M., Steedman, S., Ward, S., Winickoff, D., & Philp, J. (2019). Enabling the advanced bioeconomy through public policy supporting biofoundries and engineering biology. *Trends in Biotechnology*, *37*(9), 917–920. 31036350 10.1016/j.tibtech.2019.03.017

[CR43] Kitney, R., & Freemont, P. (2012). Synthetic biology - the state of play. *FEBS Letters*, *586*(15), 2029–2036. 22704968 10.1016/j.febslet.2012.06.002

[CR45] Kranzberg, M. (1964). Technology and human values. *The Virginia Quarterly Review*, *40*, 578–592.

[CR46] Lamine, W., Mian, S., Fayolle, A., Wright, M., Klofsten, M., & Etzkowitz, H. (2018). Technology business incubation mechanisms and sustainable regional development. *Journal of Technology Transfer*, *43*, 1121–1141. 10.1007/s10961-016-9537-9

[CR47] Latapí Agudelo, M. A., Jóhannsdóttir, L., & Davídsdóttir, B. A. (2019). Literature review of the history and evolution of corporate social responsibility. *International Journal of Corporate Social Responsibility*, 4(1).

[CR48] Lesaffre (2022). Lesaffre inaugurates its Campus and announces its R&D ambitions to accelerate innovation. 13 October. https://www.lesaffre.com/press-room/lesaffre-inaugurates-its-campus-and-announces-its-rd-ambitions-to-accelerate-innovation/

[CR49] Link, A. N., Siegel, D. S., & Wright, M. (Eds.). (2015). *The Chicago Handbook of University Technology Transfer and academic entrepreneurship*. University of Chicago Press.

[CR50] Mao, N., Aggarwal, N., Poh, C. L. (2021). Future trends in synthetic biology in Asia. *Advanced Genetics*, 2 (1), e10038. 10.1002/ggn2.10038 PMC9744534 36618442

[CR52] Matthews, N. E., Cizauskas, C. A., Layton, D. S., Stamford, L., & Shapira, P. (2019a). Collaborating constructively for sustainable biotechnology. *Scientific Reports*, *9*, 19033. 31836745 10.1038/s41598-019-54331-7 PMC6910968

[CR51] Matthews, N. E., Stamford, L., & Shapira, P. (2019b). Aligning sustainability assessment with responsible research and innovation: Towards a framework for constructive sustainability assessment. *Sustainable Production and Consumption*, *20*, 58–73. 32051840 10.1016/j.spc.2019.05.002 PMC6999670

[CR53] McKinsey (2020). The Bio Revolution: Innovations transforming economies, societies, and our lives. May 13. https://www.mckinsey.com/industries/life-sciences/our-insights/the-bio-revolution-innovations-transforming-economies-societies-and-our-lives

[CR54] McManus, M. C., Taylor, C. M., Mohr, A., Whittaker, C., Scown, C. D., Borrion, A. L., Glithero, N. J., & Yin, Y. (2015). Challenge clusters facing LCA in environmental decision-making—what we can learn from biofuels. *International Journal of Life Cycle Assessment*, *20*, 1399–1414. 27453635 10.1007/s11367-015-0930-7 PMC4939404

[CR55] Molloy, J., Felipe, J., Morales, M., Kuroshenkova, A., & Kutschera, A. (2018). Microbial Bioreactor. Team Open Bioeconomy Lab, https://www.hackster.io/open-bioeconomy-lab/microbial-bioreactor-d7f61b [Accessed 30 June 2022].

[CR56] NASEM. (2020). *Safeguarding the Bioeconomy. National Academies of Sciences, Engineering, and Medicine*. The National Academies Press. https://www.nap.edu/catalog/25525/safeguarding-the-bioeconomy. 32352690

[CR57] NSF (2023). BioFoundries to Enable Access to Infrastructure and Resources for Advancing Modern Biology and Biotechnology. Alexandra, VA: National Science Foundation, Program Solicitation 23–585. https://www.nsf.gov/pubs/2023/nsf23585/nsf23585.htm

[CR58] OECD. (2009). *The Bioeconomy to 2030: Designing a Policy Agenda. Main findings and policy conclusions*. Organisation for Economic Cooperation and Development.

[CR59] OECD (2011). Industrial Biotechnology and Climate Change: Opportunities and Challenges. https://www.oecd.org/sti/emerging-tech/49024032.pdf (Accessed: 20 May 2023).

[CR60] OECD. (2021). Accelerating innovation to meet global challenges: The role of engineering biology. *OECD Science, Technology and Innovation Outlook 2021, chap. 7*. Organisation for Economic Cooperation and Development.

[CR61] Owen, R., Pansera, M., Macnaghten, P., & Randles, S. (2020). Organisational institutionalisation of responsible innovation. *Research Policy*, *50*(1), 104132. 10.1016/j.respol.2020.104132

[CR62] Palmeros Parada, M., Osseweijer, P., & Posada Duque, J. A. (2017). Sustainable biorefineries, an analysis of practices for incorporating sustainability in biorefinery design. *Industrial Crops and Products*, *106*, 105–123. 10.1016/j.indcrop.2016.08.052

[CR63] Pansera, M., Owen, R., Meacham, D., & Kuh, V. (2020). Embedding responsible innovation within synthetic biology research and innovation: Insights from a UK multi-disciplinary research centre. *Journal of Responsible Innovation*, *7*(3), 384–409. 10.1080/23299460.2020.1785678

[CR64] Patton, M. Q. (2002). Qualitative research and evaluation methods. 3rd edition. Sage Publications, Thousand Oaks, CA.

[CR65] RAE. (2019). *Engineering Biology: A Priority for Growth*. Royal Academy of Engineering.

[CR66] Rossi, F., Caloffi, A., Colovic, A., & Russo, M. (2022). New business models for public innovation intermediaries supporting emerging innovation systems: The case of the internet of things. *Technological Forecasting and Social Change*, *175*, 121357. 10.1016/j.techfore.2021.121357

[CR67] Rossi, F., Colovic, A., Caloffi, A., & Russo, M. (2021). Public innovation intermediaries and digital co-creation, Working Paper 50, Birkbeck Centre for Innovation Management Research, revised Feb 2021.

[CR81] Russo, M., Caloffi, A., Rossi, F., & Righi, R. (2019). Innovation intermediaries and performance-based incentives: A case study of regional innovation poles. *Science and Public Policy*, *46*(1), 1–12.

[CR68] SBRCG. (2012). *A Synthetic Biology Roadmap for the UK. UK Synthetic Biology Roadmap Coordination Group*. Technology Strategy Board.

[CR69] Serrano, L. (2007). Synthetic biology: Promises and challenges. *Molecular Systems Biology*, *3*(1), 158. 18091727 10.1038/msb4100202 PMC2174633

[CR70] Shapira, P., & Kwon, S. (2018). Synthetic Biology Research and Innovation Profile 2018. Publications and Patents. bioRxiv. 10.1101/485805

[CR71] Shapira, P., & Youtie, J. (2017). Institutions for Technology Diffusion and the Next Production Revolution. In: OECD, The Next Production Revolution: Implications for Governments and Business, Organisation for Economic Cooperation and Development, Paris. 10.1787/9789264271036-en, 243–275.

[CR72] Siegel, D. (2006). *Technological Entrepreneurship: Institutions and agents involved in University Technology Transfer*. Edward Elgar Publishing.

[CR73] Siegel, J., & Krishnan, S. (2020). Cultivating invisible impact with deep technology and creative destruction. *Journal of Innovation Management*, *8*(3), 6–19. 10.24840/2183-0606_008.003_0002

[CR74] Stilgoe, J., Owen, R., & Macnaghten, P. (2013). Developing a framework for responsible innovation. *Research Policy*, *42*, 1568–1580. 10.1016/j.respol.2013.05.008

[CR75] Swedberg, R. (2020). Exploratory research. In C. Elman, J. Gerring, & J. Mahoney (Eds.), *The production of knowledge: Enhancing Progress in Social Science* (pp. 17–41). Cambridge University Press.

[CR76] Thakur, S., & Raghunathan (2021). Biofoundries: The next frontier in cell factory design and manufacturing. *Asian Biotechnology and Development Review*, *23*(3), 81–100.

[CR77] Varjani, S. J., Parameswaran, B., Kumar, S., & Khare, S. K. (2018). *Biosynthetic Technology and Environmental challenges*. Springer Nature.

[CR78] Vidmar, M. (2020). What are innovation intermediaries? *Innovation intermediaries and (final) frontiers of high-tech*. Palgrave Macmillan. 10.1007/978-3-030-60642-8_2

[CR79] von Schomberg, R. (2013). A vision of responsible Research and Innovation. In R. Owen, J. Bessant, & M. Heintz (Eds.), *Responsible Innovation*. John Wiley & Sons, Ltd.

[CR80] Youtie, J., Ward, R., Shapira, P., Schillo, R. S., & Earl, E. L. (2021). Exploring new approaches to understanding innovation ecosystems. *Technology Analysis & Strategic Management*, *35*(3), 255–269. 10.1080/09537325.2021.1972965

